# Supplementary material for: Weyl-like points from band inversions of spin-polarised surface states in NbGeSb
Source: Nat Commun. 2019 Dec 2;10:5485. doi: 10.1038/s41467-019-13464-z (PMC6888910; doi:10.1038/s41467-019-13464-z)
Supplement: Supplementary file 2 — Supplementary Information [file 41467_2019_13464_MOESM2_ESM.pdf]

Supplementary Information

Weyl-like points from band inversions of spin-polarised surface states in NbGeSb

Marković *et al.*

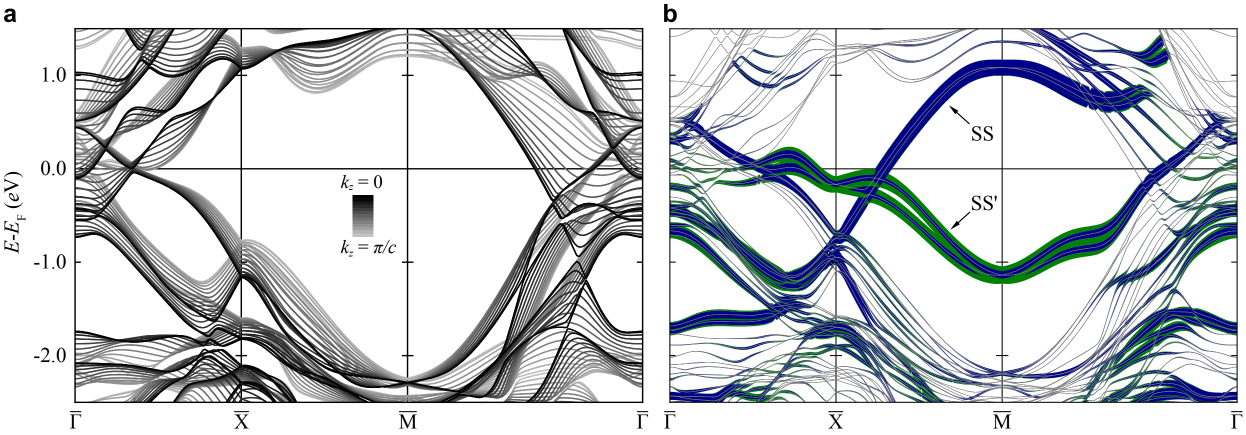

Supplementary Figure 1. **Bulk and surface calculated band structure of NbGeSb.** A side-by-side comparison of (a) a series of k_z-projected bulk DFT band structure calculations and (b) the DFT surface slab calculation reproduced from Fig. 1 of the main text. The grey colours in (a) indicate the value of the out-of-plane momentum, while line colours and weight in (b) represent the wavefunction projection onto the surface Nb (blue) and Sb (green) atoms, indicating the surface character of the states. From comparison of the two calculations, it is clear that SS and SS′ are well-defined surface states where they disperse along the $\overline{\mathrm{X}}-\overline{\mathrm{M}}$ line, but become resonant with the bulk states in other portions of the Brillouin zone (e.g. close to $\overline{\Gamma}$).

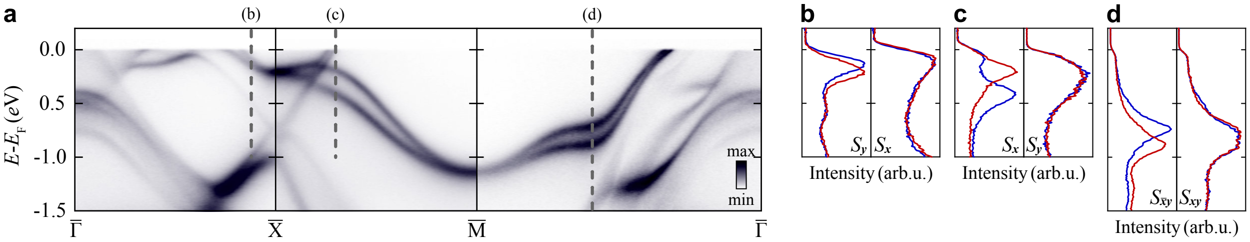

Supplementary Figure 2. **Spin polarisation of surface states along the high-symmetry lines of NbGeSb.** (a) ARPES dispersions along the high-symmetry lines of the surface projected Brillouin zone of NbGeSb (as in Fig. 1(a) of the main text) with indicated positions where spin-resolved data was taken. (b-d) Spin-polarised energy distribution curves (EDC) along the lines indicated in the dispersions. For each cut we show spin-resolved data for the spin component perpendicular and parallel to the corresponding high-symmetry line. We observe a complete extinction of the surface state spin component parallel to the high-symmetry line as per the symmetry considerations presented in the main text. A consequence of these symmetry constraints is that these surface states must necessarily have a spin polarisation of 100% away from the hybridisation points where small band gaps open. From fitting our experimental EDCs, we extract spin polarisations of ca. 70-90% for the surface bands under study here. While this is lower than the complete spin polarisation necessitated by symmetry, we note that this is a multi-orbital system, of the form where inter-orbital photoelectron interference processes are known to influence the quantitative degree of spin polarisation that is observed in a SARPES experiment[1]. Consequently, our experimental measurements are consistent with the conclusion of 100% spin polarisation in the initial states that is assumed in our modelling utilised in the main text, as also supported by our *ab initio* calculations.

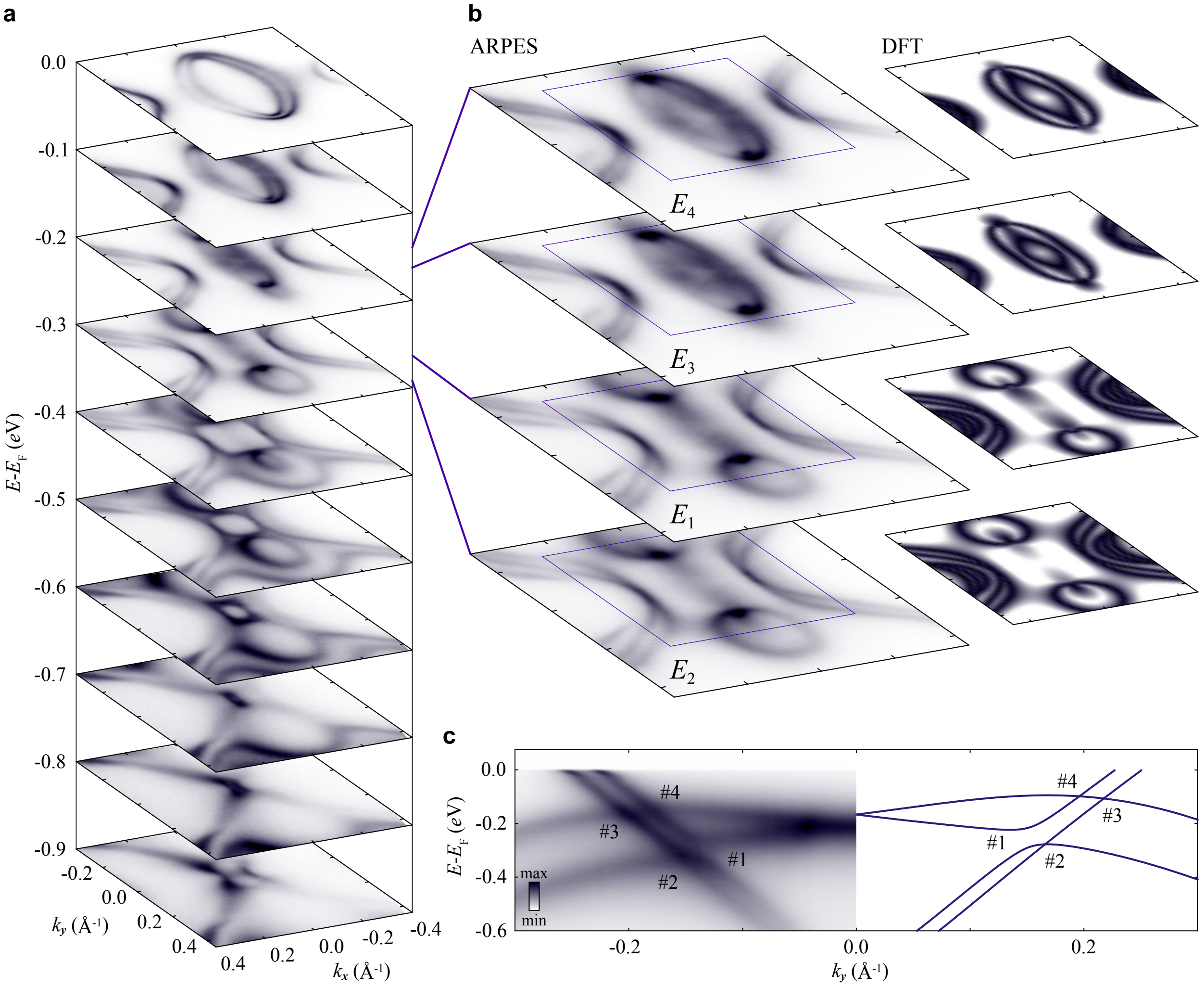

Supplementary Figure 3. **Surface state electronic structure around $\overline{\mathrm{X}}$.** (a) Constant energy contours showing the evolution of the surface state Fermi pockets at $\overline{\mathrm{X}}$ with binding energy. (b) Selected constant energy contours shown at the binding energies $E_1 - E_4$ of the four points (#1−#4) where the SS and SS′ surface states cross along the Brillouin zone edge as determined from ARPES experiments (left) and DFT calculations (right). (c) The surface state band structure along the $\overline{\mathrm{X}}$–$\overline{\mathrm{M}}$ line, showing the four band crossings in question in a measured ARPES dispersion (left) and DFT calculation (right).

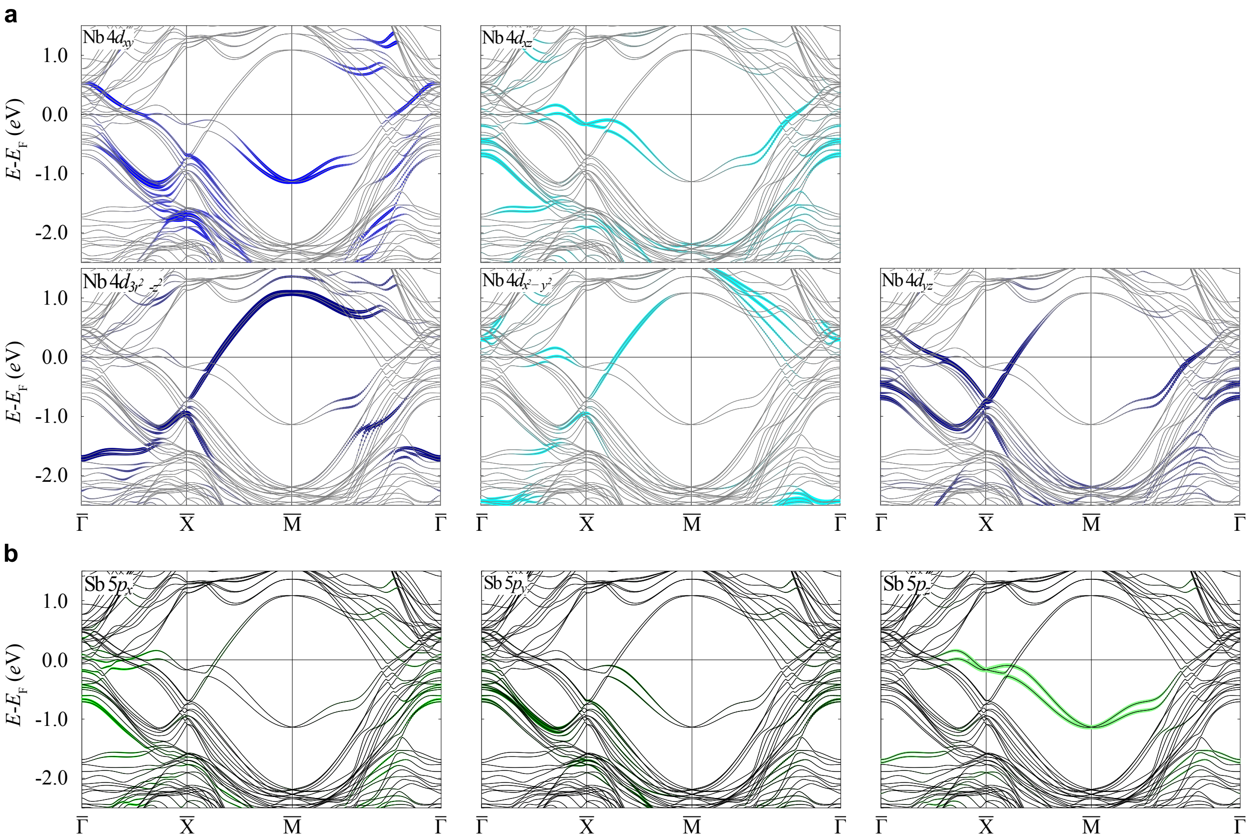

Supplementary Figure 4. **Orbital character of the surface states.** Surface slab calculations of the electronic structure from density-functional theory along the high-symmetry lines, projected onto (a) the surface Nb 4d-orbital manifold and (b) the Sb 5p-orbital manifold. The existence of the M_x mirror symmetry means that any non-degenerate state on the relevant $\overline{\text{X}}$–$\overline{\text{M}}$ line must be an eigenstate of that symmetry, i.e. either even or odd under the transformation $x \to -x$. Nb orbital content of the bands (a) along the $\overline{\text{X}}$–$\overline{\text{M}}$ line clearly satisfies this requirement: SS contains only those orbitals that are even under M_x, viz. $d_{3z^2-r^2}$, $d_{x^2-y^2}$, and d_{yz} (bottom row), while SS$'$ contains only those that are odd, viz. d_{xy} and d_{xz} (top row). SS$'$ also has significant Sb p_z-orbital content. Since p_z is even under M_x, that would appear to violate the above rule. It does not, however, because of additional Bloch phase factors that are incurred when M_x acts on the Sb orbitals. These arise because there is no M_x mirror plane that contains both the Nb and Sb atoms. Therefore, if we choose a mirror plane that leaves the Nb sites undisturbed, it will necessarily translate the Sb sites, and a compensating translation — with the associated Bloch factor $e^{i\mathbf{k}\cdot\mathbf{r}}$ — is necessary to bring them back to their original positions. The translation vector is $\mathbf{r} = (a, 0)$, where a is the Bravais lattice spacing; and on the $\overline{\text{X}}$–$\overline{\text{M}}$ line we have $k_x = \pi/a$. Therefore the Bloch factor is $e^{i\pi} = -1$, which means that those orbitals of Sb that are naïvely even under M_x are in fact odd, and vice versa.

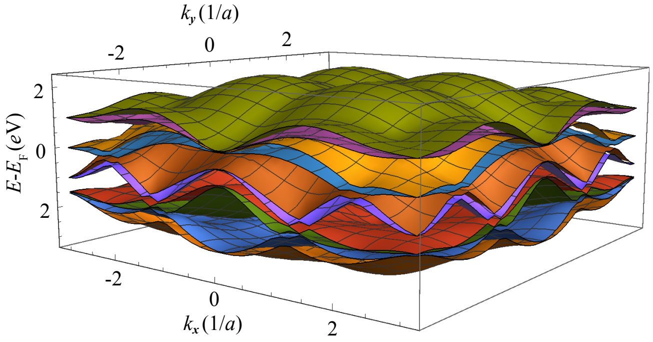

Supplementary Figure 5. **Full 5-orbital tight-binding model.** The tight-binding model was based on the five Nb 4*d* orbitals including spin. While relevant crystalline symmetries impose restrictions to certain hopping integrals of the Hamiltonian, the others were chosen arbitrarily, setting the spin-independent part of the Hamiltonian. We then add a Rashba spin-orbit coupling term, and finally an intra-unit-cell spin-orbit coupling term, $\mathbf{L} \cdot \mathbf{S}$. The model has spectator bands in the same energy range as the relevant surface state pairs, but still reproduces a crossing structure along the Brillouin zone edges similar to that of NbGeSb, which is reproduced in Fig. 3(a,b) and Fig. 4 of the main text. In reality, the other states of this manifold in NbGeSb are pushed further away in energy and become resonant with projected bulk bands. For more details on the tight-binding model, see the Methods section of the main text.

[1] Zhu, Z.-H. *et al.* Layer-By-Layer Entangled Spin-Orbital Texture of the Topological Surface State in Bi_2Se_3. *Physical Review Letters* **110**, 216401 (2013).
